# Who’s cooking? Trends in US home food preparation by gender, education, and race/ethnicity from 2003 to 2016

**DOI:** 10.1186/s12937-018-0347-9

**Published:** 2018-04-02

**Authors:** Lindsey Smith Taillie

**Affiliations:** Department of Nutrition, Gillings School of Global Public Health, and the Carolina Population Center, University of North Carolina, CB # 8120 University Square, Chapel Hill, North Carolina, 27514 USA

**Keywords:** Cooking, Home food preparation, Socio-economic status, Education, Race/ethnicity, Disparities, American Time Use Survey

## Abstract

**Background:**

While US home cooking declined in the late twentieth century, it is unclear whether the trend has continued. This study examines home cooking from 2003 to 2016 by gender, educational attainment, and race/ethnicity.

**Methods:**

Nationally representative data from the American Time Use Study from 2003 to 2016 and linear regression models were used to examine changes in the percent of adults aged 18–65 years who cook and their time spent cooking, with interactions to test for differential changes by demographic variables of gender, education, and race/ethnicity.

**Results:**

Cooking increased overall from 2003 to 2016. The percent of college-educated men cooking increased from 37.9% in 2003 to 51.9% in 2016, but men with less than high school education who cook did not change (33.2% in 2016) (*p* < 0.05). College-educated women who cook increased from 64.7% in 2003 to 68.7% in 2016, while women with less than high school education had no change (72.3% in 2016) (*p* < 0.05). Women with less education spent more time cooking per day than high-educated women, but the reverse was true for men. Among men, the percent who cook increased for all race/ethnic groups except non-Hispanic blacks. Among women, only non-Hispanic whites increased in percent who cook. Among both men and women, non-Hispanic blacks had the lowest percentage who cooked, and non-Hispanic others spent the greatest amount of time cooking.

**Conclusions:**

Home cooking in the United States is increasing, especially among men, though women still cook much more than men. Further research is needed to understand whether the heterogeneity in home cooking by educational attainment and race/ethnicity observed here contributes to diet-related disparities in the United States.

## Background

In recent years, scholars [1] and celebrities alike [2, 3] have called for a return to home cooking as a key strategy to improve dietary intake and prevent obesity. Growing, but limited, evidence suggests that cooking from scratch has many benefits. Intervention studies on improving cooking skills have shown increased cooking confidence, frequency of cooking, and intake of fruits and vegetables [4–6]. Other studies have also shown that cooking skill is associated with lower intakes of ultra-processed food, convenience food, and take-away food [7, 8]. In addition, a recent systematic review found that increased home cooking is associated with overall healthier dietary patterns [9], though authors noted that many studies on cooking are observational and thus required stronger evidence.

Despite the potential benefits of home cooking on dietary intake, overall cooking levels in the US declined in the second half of the twentieth century and early years of the twenty first century [10], with increased food costs, decreased time availability, and lack of skill noted as key factors [11–13]. Though these barriers seem to have persisted through the last decade, interest in cooking for leisure or entertainment has increased dramatically. This is evident in the increasing popularity of food-related media such as food-focused television channels [14], celebrity chefs [15, 16], food magazines, cookbooks, and blogs, as well as digital [17] and social media [18] and smartphone applications focused on cooking [17], suggesting changes in social norms and values around cooking [19]. Thus, one question is whether the decades-long decline in home cooking has continued, or whether it has begun to plateau or even reverse.

A second question is who does the home cooking. While women have traditionally been the predominant food shoppers and preparers [20], some evidence suggests this is beginning to shift, with men taking an increasing role [10]. It is also important to understand whether trends in home cooking differ by education level or race/ethnicity, as individuals of lower socio-economic status and racial/ethnic minorities are more likely to have poorer diet quality and suffer from diet-related diseases such as obesity and type 2 diabetes [21–23].

The objective of this study was to describe trends in the percent of individuals who cooked as well as the amount of time spent cooking (min/capita/day) from 2003 to 2016 using nationally representative data on time use, by gender, education level, and race/ethnicity.

## Methods

This study analyzed public-use data from the American Time Use Survey (ATUS) with no personally identifiable information. No *institutional review board* approval was required.

Details on the ATUS have been published extensively [24]. Data were downloaded from the ATUS Extract Builder Database in 2017 (https://www.atusdata.org/atus/) and are publicly available [25]. ATUS has been conducted annually by the US Census Bureau since 2003, with the goal to develop nationally representative estimates of time use for Americans. The sample consists of randomly selected households who have completed their final interview in the Current Population Survey. Within each household, one individual over age 15 is randomly selected to participate. Computer-assisted telephone interviews probe respondents on time use during the previous 24-h period. Participants report activities and the duration of activities, which are then coded by ATUS staff into activity types using the ATUS Activity Coding Lexicon, which is a three-tiered classification system [26]. The current analysis includes pooled data from 2003 to 2016 for adults aged 18 to 65 years on non-holidays. Outliers were excluded for reported cooking time (> 8.5 h/day), representing the top 0.1% of the distribution. The final analytical sample included 139,219 respondents.

### Outcome measures

In this study, “home cooking” refers to the sum of reported time spent in all activities that were categorized as food and drink preparation (e.g., baking, cooking, broiling, boiling; packing lunches; heating up food), food presentation (setting the table, filling salt and pepper shakers, serving the meal), kitchen and food clean-up (e.g., clearing the table, washing dishes, storing leftovers), and other food preparation activities not otherwise specified.

### Explanatory measures

Covariates included survey year, respondents’ gender, age, race/ethnicity, educational level, employment status, and marital status, and presence of one or more children under 18 years of age. Gender was self-reported as male or female. Race/ethnicity was categorized as non-Hispanic white, non-Hispanic black, Hispanic, or other. As in previous work [27], age was categorized into three groups to reflect progression from early adulthood to retirement ages: 18–29 years, 30–44 years, or 45–65 years. Education was also categorized into three groups: less than high school, high school degree or some college, or college degree or higher. Employment status was defined as: not in the labor force (retired, unemployed, or other), part-time (< 35 h/week), or full-time (≥35 h/week). Marital status was defined as either married (having a spouse or unmarried partner) or single.

### Statistical analysis

Stata version 14.2 (StataCorp LP, College Station, TX) was used for all statistical analyses. All analyses were weighted to be nationally representative.

Descriptive statistics (proportions testing or t-tests) were used to examine changes in the proportion of respondents who reported home cooking and mean time spent home cooking from 2003 to 2016 (min/per capita/day), by socio-demographic characteristics.

Linear regression models were used to examine changes in the adjusted predicted proportion of respondents who home cooked and the mean per capita time spent cooking from 2003 to 2016, controlling for gender, age, race/ethnicity, education level, employment status, marital status, and presence of children <18y. Each covariate was included as a set of indicator variables to allow for non-linear associations. Due to the high prevalence of individuals who reported no cooking, a two-part model was used to estimate the adjusted mean time spent cooking. Separate models were used to test the interaction between gender, education, and year as well as gender, race/ethnicity, and year in order to examine whether changes in home cooking over time were different for men and women of by educational levels or race/ethnicities. A sensitivity analysis was conducted to examine the trends in the time spent in cooking among only those who reported cooking. Wald chunk test were used to determine the statistical significance of each interaction. The margins command in Stata was used to estimate adjusted predicted proportions who cooked or mean time spent cooking. *P*-values of < 0.05 were considered statistically significant.

## Results

Table 1 shows descriptive changes in the percent who cooked at home and the mean time spent cooking by socio-demographic variables in 2003 and 2016. The percent of men who home cooked increased more from 2003 to 2016 than for women (+ 9% vs. + 3%, respectively), although a greater percent of women still cooked in 2016 than did men (70% vs. 46%). While the mean per capita time spent cooking increased over time for both men and women, women cooked for a much greater amount of time than did men (50 min/capita/day vs. 20 min/capita/day).

**Table 1:** Descriptive trends in the percent who cooked and mean per capita time spent cooking, 2003 to 2006 (*N* = 139,219)

	N=		% who cooked at home			Mean per capita time spent cooking (min/person/day)				
	2003	2016	2003	2016	*p*-value	2003	SE	2016	SE	*p*-value
Gender										
Male	7243	3508	35%	46%	0.000	15.4	0.5	20.4	0.8	0.000
Female	9104	4199	67%	70%	0.006	46.6	0.8	49.5	1.2	0.045
Race/Ethnicity										
NH white	11,726	4791	52%	60%	0.000	29.3	0.5	32.3	0.9	0.006
NH black	1892	1124	48%	50%	0.529	29.3	1.4	32.2	2.0	0.229
Hispanic	1960	1301	51%	58%	0.006	42.2	1.8	41.9	2.0	0.902
NH other	769	491	49%	62%	0.000	33.2	2.2	52.3	3.9	0.000
Education										
< HS	1866	683	49%	53%	0.121	38.2	1.7	43.3	2.9	0.124
HS or Some College	9385	3970	51%	57%	0.000	30.4	0.6	34.3	1.1	0.002
College or greater	5096	3054	53%	61%	0.000	29.1	0.7	33.7	1.1	0.000
Age group										
18–25	2874	1224	39%	48%	0.000	21.1	0.9	24.3	1.5	0.063
26–40	6641	2804	55%	62%	0.000	35.6	0.8	40.9	1.4	0.001
41–65	6832	3679	56%	62%	0.000	33.9	0.8	37.9	1.1	0.004
Employment status										
Not in labor force	4463	2131	63%	67%	0.019	49.0	1.2	54.9	1.9	0.008
Working part-time	2107	996	55%	59%	0.227	33.5	1.3	34.6	2.0	0.639
Working full-time	9777	4580	45%	54%	0.000	22.6	0.5	26.3	0.8	0.000
Marital status										
Unmarried	6038	3356	43%	51%	0.000	21.6	0.6	25.9	1.0	0.000
Married	10,309	4351	56%	63%	0.000	36.7	0.6	41.8	1.1	0.000
Child <18y present										
No children	8805	4317	46%	53%	0.000	24.9	0.5	28.1	0.9	0.002
1 or more children	7542	3390	60%	68%	0.000	41.7	0.8	48.9	1.3	0.000

*NH* non-Hispanic, *HS* high school

There were not marked differences in the percent who cooked by race/ethnicity in 2003 or 2016. The Non-Hispanic other group showed the greatest change in amount of time spent cooking and in 2016 spent the most time cooking of any race/ethnic group. Those who were older (26–40 or 41–65), married, not in the labor force, or who had at least one child < 18 were more likely to cook and cooked for a greater amount of time, and this remained consistent over time.

**Fig. 1:**
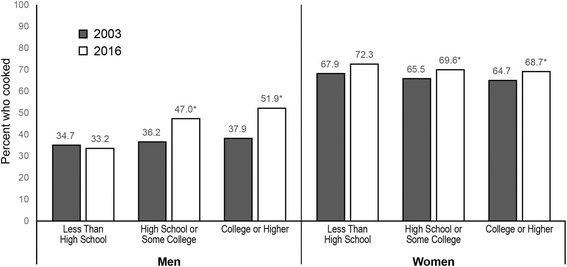
Trends in adjusted predicted percent who cooked by gender and education, 2003 to 2016. Legend: * *p* < 0.05 for 2003 vs. 2016 adjusted predicted percent, within gender and education level

When considering adjusted models, men saw larger increases in the percent who cooked from 2003 to 2016 than women, with greater changes among men with more education (*p* < 0.01 for interaction) (Fig. 1). In 2016, 10.8% more men with a high school degree or some college and 14.0% more men with a college degree cooked compared to 2003 (*p* < 0.01 for both comparisons), whereas there was no change in the percent of men without a high school degree who cooked. For women, the increase in the percent cooking from 2003 to 2016 was smaller (4.1% for both women with a high school degree or some college and women with a college degree, no change for women without a high school degree). However, the overall percent of women who cooked was much higher and less variable by education level than it was for men.

**Fig. 2:**
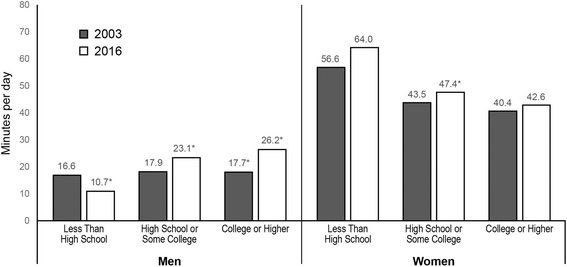
Trends in adjusted predicted mean time spent cooking by gender and education, 2003 to 2016. Legend: * *p* < 0.05 for 2003 vs. 2016 adjusted predicted mean, within gender and education level

There was also heterogeneity by gender and education with regards to changes in time spent cooking in 2003 versus 2016 (*p* < 0.01 for interaction) (Fig. 2). Among men, those with college degrees increased mean cooking time the most (+ 8.4 min/capita/day, *p* < 0.01), followed by men with a high school degree or some college (+ 5.2 min/capita/day, *p* < 0.01). Men without a high school degree actually decreased mean time spent cooking (− 5.9 min/capita/day, *p* < 0.05) heightening the disparity in time men spent cooking by education level in 2016. Among women, only those with a high school degree or some college significantly changed the amount of time spent cooking between 2003 and 2016 (+ 3.9 min/capita/day, *p* < 0.05).

**Fig. 3:**
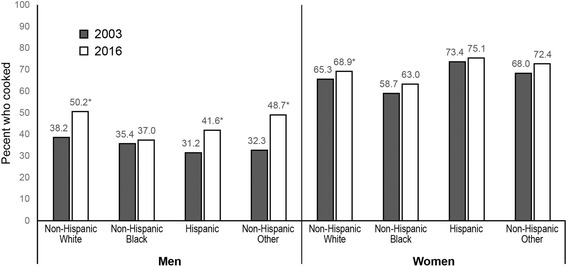
Trends in adjusted predicted percent who cooked by gender and race/ethnicity, 2003 to 2016. Legend: * *p* < 0.05 for 2003 vs. 2016 adjusted predicted percent, within gender and race/ethnicity

**Fig. 4:**
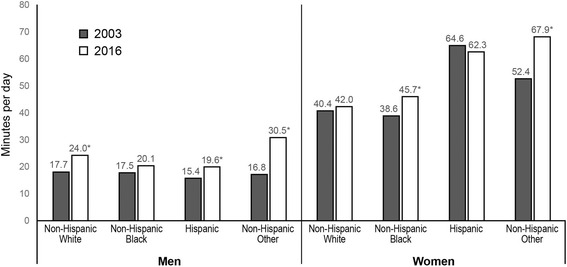
Trends in adjusted predicted mean time spent cooking by gender and race/ethnicity, 2003 to 2016. Legend: * *p* < 0.05 for 2003 vs. 2016 adjusted predicted mean, within gender and race/ethnicity

Percent who cooked and mean time spent cooking differed by race/ethnicity (*p* < 0.01 for both interactions) (Figs. 3 and 4). Among men, non-Hispanic others showed the largest increase from 2003 to 2016 in the percent who cooked (+ 16.4%), followed by non-Hispanic whites (+ 12.0%), and Hispanics (+ 10.4%) (*p* < 0.01 for each comparison). Non-Hispanic black men saw no significant change in percent who cooked from 2003 to 2016. As a result, by 2016, there was substantial heterogeneity in the percent of men who cooked by race/ethnicity, with about half of non-Hispanic white and non-Hispanic other men cooking, 42% of Hispanic men cooking, and only 37% of non-Hispanic black men cooking. Figure 4 shows that non-Hispanic other men also showed the biggest increase in mean time spent cooking (+ 13.7 min/day, *p* < 0.01), followed by non-Hispanic white (+ 6.2 min/capita/day, *p* < 0.01) and Hispanic men (+ 4.2 min/ capita/day, *p* < 0.01), whereas non-Hispanic black men had no significant change from 2003 to 2016.

Among women, on the other hand, only non-Hispanic white women showed increases in the percent who cooked (+ 3.6%, *p* < 0.05) from 2003 to 2016, although Hispanic and non-Hispanic women had the highest percent of women who cooked across all time periods (68–75%), while the smallest percent of black women cooked across time. With regards to time spent cooking, non-Hispanic other women showed the biggest increase from 2003 to 2016 (+ 15.5 min/capita/day, *p* < 0.05), and non-Hispanic black women also increased (+ 7.2 min//capita/day, *p* < 0.05), whereas non-Hispanic white women and Hispanic women showed no change. As a result, in 2016, non-Hispanic other and Hispanic women reported the highest mean time spent cooking (62.3 and 67.9 min/capita/day, respectively), whereas non-Hispanic white and black women reported lower mean time spent cooking (42.0 and 45.7 min/capita/day, respectively).

Results of the sensitivity analyses examining trends in time spent cooking only amongst those who cooked can be found in Appendix. Differences over time by gender, education, and race/ethnicity tended to be in the same direction and magnitude as the per capita estimates, but changes over time were less likely to be statistically significant.

## Discussion

This study finds that home cooking in the United States appears to be on the rise, both in terms of the percent of the US population who cooks and — except for men with less than high school education — the amount of time spent cooking. For men, this continues the trend towards increased home cooking. A previous study found that the percent of men who cooked increased from 29% in 1965 to 42% in 2007 [10], and current results show a further increase to 46% in 2016. For women, these findings indicate a leveling off or even a reversal of previous trends, as the percent of women who cooked decreased from 92% in 1965 to 68% in 2007 [10], but rebounded slightly to 70% in 2016.

### Gender

The increase in men’s cooking found here mirrors trends in Europe, where research from four Nordic countries found increases from 1997 to 2012 in the percent of men cooking, particularly those from the working and upper classes [28]. Even in 2016, however, US males’ cooking levels were still lower than in the United Kingdom a decade earlier, when a 2005 time use survey showed 60% of men (and 85% of women) in the United Kingdom cooked over one 24-h period [29]. It is unclear what accounts for this increase in US males’ home cooking, although one possibility is that the rise in popularity of food-related media has disproportionately influenced men. For example, one study found that watching cooking programs was associated with more cooking only among men [30], though additional research has found that only 28% of adults learned to cook by watching cooking shows (with no difference by gender) [19]. Others suggest that popular celebrity chefs such as Jamie Oliver have presented cooking as a masculine activity [31], potentially making it more appealing to males. At the same time, this masculinization seems to have arisen as part of “foodie culture,” or the treatment of cooking as a form of leisure or entertainment rather than labor [31–33].

Of course, having the time, money, and skill to cook as a luxury rather than a necessity is likely only possible for the middle- or upper-class. This could explain why the current study found increases in cooking only for middle- or higher-educated men, but no change for lower educated men. Increased enjoyment from cooking for men could also have contributed to increased cooking levels, as enjoyment of cooking has been linked to more cooking [34]. Interestingly, cooking research from the UK and France shows that socio-economic factors are unrelated to time spent cooking for men [29, 35].

Despite their greater increases in home cooking over time, men still lag behind women in terms of the percent who cook and time spent cooking, suggesting that women remain the primary home food preparers in the United States. The reason for the small increases in women’s home cooking, which mark a reversal of previous trends, and continued relatively high levels of cooking is not entirely clear. One possibility is that women — or at least some women — have more time available for cooking due to small declines in time spent working. Women’s labor force participation, which increased in the United States during the second half of the twentieth century, has actually fallen by about 3.5 percentage points since 2000 [36]. Less time spent in the labor force could increase time available for home cooking, which has been well-documented as a major barrier to home food preparation [11, 37, 38]. Plateaus or increases in women’s cooking time could also reflect fewer new advances in time-saving technology in the kitchen (e.g., microwaves, food processors, dishwasher) over recent years compared to the late twentieth century. In addition, strong social norms likely persist around gender and cooking: women and girls are more likely to be involved in cooking, feel confident in cooking, and pass down cooking skills to children [9]. Additional evidence shows that cooking skills and mealtime practices in general also tend to be transferred from mothers to daughters [39, 40], further propagating this norm.

### Education level

There was substantial heterogeneity in cooking trends by education level. This was especially pronounced for men: while the percent of men with high school/some college and college degrees who cooked increased to 47.0% and 51.9%, respectively, the percent of men with less than a high school degree who cooked remained low (about a third). A similar trend was observed in time spent cooking, with more educated men increasing cooking time from 2003 to 2016, while those with less than a high school degree decreasing cooking time. More cooking among higher-educated men is consistent with evidence showing that higher education is associated with more egalitarian ideas about gender roles, including more equitable distributions of household labor [41]. Among men with less education, lower cooking prevalence and time spent cooking suggests greater reliance on away-from-home foods such as fast food or restaurant food, or more frequent use of foods that are faster to prepare, such as ready-to-heat and ready-to-eat convenience foods. This could be problematic for diet quality and health, as highly processed convenience foods tend to be energy-dense and contain higher levels of added sugar, saturated fat, and sodium [42–44], whereas more time spent on cooking is associated with higher intakes of beneficial foods such as vegetables, salads, and fruits [45].

Among women, this difference was reversed: women with less than a high school education were more likely to cook and cooked for longer than those with higher levels of education. This contrasts with findings from a previous study using the National Health and Nutrition Survey, which found no association between education and likelihood of being the main meal planner or preparer [46]. That study, however, asked respondents only about their status as the “main” meal preparer and not about their likelihood to cook or the amount of time they spent cooking. Interestingly, the present study found no increase in either measure of cooking for low-educated women from 2003 to 2016. This is somewhat surprising, given that economic changes such as the Great Recession, increases in food prices, and wage stagnation might suggest greater increases in cooking as people trade time to save money. These findings are consistent, however, with those in a previous study, which found that low socio-economic status households (as measured by the poverty rate) did not alter cooking patterns in response to the Great Recession [27].

It is worth noting that more cooking does not necessarily equate to more healthful cooking. Some studies have shown that low-socio-economic households have lower levels of confidence in cooking from scratch or cooking with vegetables [13, 47], and may be likely to rely on ready-to-eat meals or frozen convenience foods or fried foods. Research has shown that people in low-income/low-access neighborhoods have noted food affordability — particularly for fresh produce and other basic ingredients — as major barrier to buying and preparing healthier foods [34]. In fact, one recent study found that women who spent more time preparing meals actually had greater risk of metabolic syndrome [48]. While the current work describes trends in home cooking, more research is needed to link these shifts in dietary behaviors to changes in dietary intake and downstream effects on obesity and cardio-metabolic risk.

### Race/ethnicity

There was a high level of heterogeneity in home cooking by race/ethnicity, as well, particularly for men. While non-Hispanic other men had the greatest increase in percent who cooked, a similarly high percent of white men cooked in 2016, followed by Hispanic men, and these trends were consistent for amount of time spent cooking. Non-Hispanic black men were the only race/ethnic group that did not increase cooking time from 2003 to 2016, and in 2016 spent the least time cooking of any group. For women, on the other hand, both the relative change over time as well as absolute differences between race/ethnic groups were much smaller with regards to the percent cooking, though again, non-Hispanic blacks had the lowest levels. However, non-Hispanic white women spent the least amount of time cooking in 2016, followed closely by black women, and Hispanic and non-Hispanic other women spent substantially more time cooking (> 20 min/day compared to non-Hispanic whites). The low level of cooking among non-Hispanic black men and women as well as higher levels of cooking among Hispanics is consistent with previous findings [49].

One question for future study is how amount of cooking interacts with ingredients and methods used to influence dietary intake and downstream health effects, as there is likely great heterogeneity in these aspects of cooking behavior as well. For example, previous research has shown that ethnicity and culture influence the ingredients used, such as the use of fresh or frozen food vs. more processed food and canned goods [50]. Other research has shown that the non-Hispanic black families may be more likely to use high levels of sugar, salt, and fat as well as less-healthy cooking methods like frying [51–53]. Given this study’s findings on relatively low levels of cooking among black men and women, more research into home food preparation in black households may be useful to understand how cooking (or lack thereof) might contribute to diet-related health disparities for black Americans.

### Limitations

This study has several limitations. First, it was not possible to examine changes in home cooking by income level due to the high level of missingness on this variable in the data. Educational attainment serves as a useful proxy for socio-economic status, has been validated as a predictor of cardiovascular risk [54], and may relate to a household’s food and nutrition-related knowledge and skill. Income plays a related but distinct role, as financial resources — or lack thereof — can limit the types of foods households can buy, whether single ingredients to cook from scratch, processed prepared foods, or foods purchased and eaten away from home. For example, research has shown that energy-dense diets high in refined grains, added sugar, and added fat cost less than fresh fruits and vegetables, meats, and fish [55], suggesting that the cost of basic ingredients (and thus home cooking) might pose an important barrier to lower-income households beyond other education-related barriers. Secondly, the time-use data is limited only to activities reported by a single individual on a single day. Thus, just because a respondent does not spend time themselves in home food preparation does not necessarily mean they are not consuming home-prepared food, either as leftovers or prepared for them by someone else. It is unclear whether dietary value differs for consuming self-prepared foods vs. consuming home-prepared food made by someone else.

One strength of the current study is that participants report all the activities in which they participated, which are then coded as “home food preparation” (or another activity) by ATUS coders. This coding structure avoids potential problems associated with variation in perceptions of what counts as home cooking by gender, education, or race/ethnicity (i.e., what one person counts as cooking, another person might not). However, the measure of home cooking used in this study does represent multiple aspects of the cooking process, from setting the table through cooking and clean-up, as well as varying levels of cooking, from simply microwaving a ready-to-eat snack to assembling several pre-prepared items to preparing an entire meal from scratch. While time spent cooking can be considered a proxy for level of cooking effort (with more time likely reflecting a more intensive preparation process or greater likelihood of being “from scratch”), it is not possible to examine this level of detail in the current study. Future research will be needed to understand which aspects of the cooking process and what level of cooking are important for better diet and health outcomes.

## Conclusions

Home cooking in the United States increased from 2003 to 2016, with greater increases among men, although women remain more likely to cook and to cook for more time. There was substantial heterogeneity in cooking behaviors by education level and race/ethnicity, with lower-educated men, higher-educated women, and non-Hispanic black men and women less likely to cook at home. These differences by education and race/ethnicity suggest that programs or policies seeking to improve diet through increased cooking may achieve the biggest gains in these sub-populations.

## Appendix

**Table 2:** Adjusted predicted mean time spent cooking (min/day) among those who cooked

	2003		2016			
	Mean	SE	Mean	SE	Diff	Contrast *p*-value^a^
Men						
Less than High School	49.4	3.7	40.2	4.0	−9.1	0.094
High School or Some College	47.8	1.3	49.1	2.0	1.3	0.593
College or Graduate School	46.6	1.4	52.1	2.2	5.5	0.036
Women						
Less than High School	81.1	3.2	85.8	4.2	4.7	0.364
High School or Some College	66.3	1.1	67.9	2.0	1.7	0.455
College or Graduate School	62.0	1.4	61.3	1.6	−0.7	0.749
Chunk test *p*-value: < 0.001						
	2003		2016			
	Mean	SE	Mean	SE	Diff	Contrast *p*-value^b^
Men						
Non-Hispanic white	45.2	1.1	48.9	1.7	3.7	0.060
Non-Hispanic black	49.4	3.9	53.0	4.2	3.6	0.533
Hispanic	50.6	3.6	47.5	2.9	−3.1	0.499
Non-Hispanic other	51.3	4.9	63.2	8.1	11.9	0.208
Women						
Non-Hispanic white	61.6	1.0	60.7	1.6	−0.8	0.666
Non-Hispanic black	66.7	2.7	72.9	3.6	6.2	0.168
Hispanic	85.8	2.9	81.4	3.1	−4.4	0.291
Non-Hispanic other	77.8	4.0	94.8	4.7	16.9	< 0.001
Chunk test *p*-value: < 0.001						

^a^Contrast for within education level, between year

^b^Contrast for within race/ethnicity and gender, between year

## Abbreviation

ATUS: American Time Use Survey
